# Expression of genes involved in brain GABAergic neurotransmission in three-spined stickleback exposed to near-future CO_2_

**DOI:** 10.1093/conphys/cow068

**Published:** 2016-12-29

**Authors:** Floriana Lai, Cathrine E Fagernes, Fredrik Jutfelt, Göran E Nilsson

**Affiliations:** 1 Section for Physiology and Cell Biology, Department of Biosciences, University of Oslo, Norway; 2 Department of Biology, Norwegian University of Science and Technology, Trondheim, Norway

**Keywords:** GABA_A_ receptor, GABAergic system, ion cotranporters, ocean acidification, quantitative polymerase chain reaction, three-spined stickleback

## Abstract

Change in the activity of the main inhibitory receptor, GABA_A_, has been suggested to be a general mechanism behind the behavioural alterations reported in ocean acidification studies on fish. It has been proposed that regulatory acid–base mechanisms in response to high CO_2_ alter the neuronal Cl^−^ and HCO_3_^−^ gradients that are important for GABA_A_ receptor function. Here, we report a comprehensive analysis of gene expression of GABA_A_ receptor subunits and of genes involved in GABAergic transmission in the brain of fish exposed to near-future CO_2_. Altogether, 56 mRNA transcripts were quantified in brains of three-spined stickleback (*Gasterosteus aculeatus*) kept in control pCO_2_ (333 ± 30 μatm CO_2_) or at high pCO_2_ levels (991 ± 57 μatm) for 43 days. The gene expression analysis included GABA_A_ receptor subunits (α1–6, β1–3, γ1–3, δ, π and ρ1–3), enzymes and transporters involved in GABA metabolism (GAD1–2, GABAT and GAT1–3), GABA_A_ receptor-associated proteins (GABARAP and GABARAPL), ion cotransporters (KCC1–4, NKCC1, ClC21–3, AE3 and NDAE) and carbonic anhydrase (CAII). Exposure to high CO_2_ had only minor effects on the expression of genes involved in GABAergic neurotransmission. There were significant increases in the mRNA levels of α family subunits of the GABA_A_ receptor, with a more pronounced expression of α1_2_, α3, α4 and α6b. No changes were detected in the expression of other GABA_A_ subunits or in genes related to receptor turnover, GABA metabolism or ion transport. Although the minor changes seen for mRNA levels might reflect compensatory mechanisms in the high-CO_2_ conditions, these were apparently insufficient to restore normal neural function, because the behavioural changes persisted within the time frame studied.

## Teaser

We report here the first comprehensive gene expression analysis of GABA_A_ receptor subunits and of genes involved in brain GABAergic transmission in fish exposed to near-future CO_2_ conditions. Altogether, 56 mRNA transcripts were quantified in brains of three-spined stickleback (*Gasterosteus aculeatus*) kept in control or elevated pCO_2_ for 43 days.

## Introduction

The ongoing increase of CO_2_ levels in the atmosphere and the resultant changes in the ocean chemistry are leading to what is commonly referred to as ocean acidification. In their most recent assessment report, the Intergovernmental Panel on Climate Change (IPCC) predicted an increase in the atmospheric CO_2_ concentration from the present level of 400 μatm to 800–1150 μatm within this century (Collins *et al*., 2013). These changes in the atmosphere can then lead to a decrease in average ocean pH of up to 0.32, with severe consequences for marine ecosystems (Doney *et al*., 2009; Ciais *et al*., 2013).

Numerous studies on ocean acidification have reported alterations in behaviour and sensory responses in both tropical and temperate fish after sustained exposure to predicted near-future CO_2_ levels. The sensory systems affected include olfaction, hearing and vision (Munday *et al*., 2009; Dixson *et al*., 2010; Ferrari *et al*., 2011; Simpson *et al*., 2011; Forsgren *et al*., 2013; Chung *et al*., 2014; Rossi *et al*., 2016). Other neural challenges detected involve brain lateralization (Domenici *et al*., 2012; Nilsson *et al*., 2012; Jutfelt *et al*., 2013; Lai *et al*., 2015), learning (Ferrari *et al*., 2012), anxiety (Hamilton *et al*., 2014), boldness and activity (Munday *et al*., 2010; Jutfelt *et al*., 2013). Nonetheless, a few studies on temperate species (Atlantic cod, *Gadus morhua*, and Atlantic silverside, *Menidia menidia*) find resilience against elevated ambient CO_2_, which can be related to adaptations in species experiencing a strong variation in the partial pressure of CO_2_ (pCO_2_) in their current habitat (Murray *et al*., 2014; Jutfelt and Hedgärde, 2015).

Studies using an antagonist (gabazine) or an agonist (muscimol) of the γ-aminobutyric acid receptor A (GABA_A_ receptor) have indicated that an altered function of this inhibitory neurotransmitter receptor underlies these behavioural abnormalities. In particular, gabazine has been found to restore much of the altered behaviours (Nilsson *et al*., 2012; Chivers *et al*., 2014; Chung *et al*., 2014; Hamilton *et al*., 2014; Lai *et al*., 2015). The GABA_A_ receptor is an ion channel with conductance for Cl^−^ and HCO_3_^−^, and these are the same two ions that are involved in pH regulation in fish exposed to elevated CO_2_. Thus, when fish are exposed to high CO_2_ levels, the reduction in blood pH is countered by accumulation of HCO_3_^−^ in blood and tissues (Ishimatsu *et al*., 2008; Brauner and Baker, 2009), accompanied by a release of H^+^ and Cl^−^ over the gills into the ambient water. This led Nilsson *et al*. (2012) to suggest that pH-regulatory changes in fish exposed to high CO_2_ alter the gradients of Cl^−^ and HCO_3_^−^ over neuronal membranes in a way that renders some GABA_A_ receptors depolarizing (i.e. excitatory) rather than hyperpolarizing (i.e. inhibitory).

The GABA_A_ receptor is the major inhibitory neurotransmitter receptor in the vertebrate brain, and ~30% of all synapses respond to GABA (Bloom and Iversen, 1971; Kaila, 1994; Somogyi *et al*., 1996; Sieghart and Sperk, 2002). It is expressed throughout the central nervous system, and its role has been linked to important processes such as brain development, neural migration and excitability, network interaction in the cerebral cortex, memory, learning, cognition, vigilance and behaviour (Sieghart and Sperk, 2002; Makkar *et al*., 2010; Luscher *et al*., 2011).

The GABA_A_ receptor is a ligand-gated ion channel composed by pentameric assemblies of subunits, arranged to form a central selective anion channel (Bormann *et al*., 1987). To date, a total of 19 genes have been found to encode for GABA_A_ receptor subunits in mammals, namely α1–6, β1–3, γ1–3, δ, π, ε, θ and ρ1–3 (reviewed by Farrant and Kaila, 2007). However, information on GABA_A_ receptor composition in fish is very scarce. An immunochemistry analysis has confirmed a widespread distribution of the receptor in Atlantic salmon (*Salmo salar*) brain (Anzelius *et al*., 1995). Ellefsen *et al*. (2008) surveyed mRNA transcripts of GABA_A_ subunits in the anoxia-tolerant crucian carp (*Carassius carassius*), quantifying the effect of anoxia on the mRNA expression of subunits α1–6, β2, γ2 and δ1–2. Cocco *et al*. (2016) recently profiled the expression of GABA_A_ subunits in zebrafish (*Danio rerio*) brain, showing α1, β2, γ2 and δ to be the most prominently expressed subunits.

The combination of different subunits in the pentameric GABA_A_ receptor can give rise to diverse receptor subtypes, with distinct physiological and pharmacological properties (Herd *et al*., 2007). Generally, a combination of the two most highly expressed subunits, α and β, is sufficient to form a functional GABA_A_ receptor, while the presence of a third subunit is also often observed (reviewed by Farrant and Kaila, 2007). Indeed, the most predominant GABA_A_ receptor stoichiometry among mammals is a heteromeric receptor composed of two α, two β and one γ subunit, with the most common combination being α1, β2 and γ2 subunits (Fritschy *et al*., 1992; McKernan and Whiting, 1996; Pirker *et al*., 2000; Sieghart and Sperk, 2002; Whiting, 2003; Benke *et al*., 2004). In other GABA_A_ receptors, the γ subunit is replaced by δ, π or ε (forming αβδ, αβπ or αβε), whereas the θ subunit might replace the β subunit (Sieghart and Sperk, 2002; reviewed by Farrant and Kaila, 2007).

Activation of the receptor takes place when two GABA molecules bind to the extracellular domains between the α and β subunit, triggering a rapid conformational change in the transmembrane region that allows movement of Cl^−^ and HCO_3_^−^ through the channel (Bormann *et al*., 1987). Intracellular and extracellular [Cl^−^] and [HCO_3_^−^] are important for setting the *E*_GABAA_ reversal potential. In most mature mammalian neurons, GABA_A_ receptor activation reduces the excitatory neurotransmission through membrane hyperpolarization caused by a net influx of negatively charged Cl^−^ ions into the neuron, with a smaller component of HCO_3_^−^ flowing out (reviewed by Farrant and Nusser, 2005). However, in the fetal mammalian brain, and in some conditions of neuronal overactivity, such as in epilepsy, anion gradients are reversed as a result of increased intracellular [Cl^−^] and/or intracellular [HCO_3_^−^] linked to a different or altered expression of ion transporters. The Cl^−^ gradient across neuronal membranes has been shown to depend largely on two ion-exchange mechanisms (Delpire, 2000). The K^+^–Cl^−^ cotransporters (KCC) are responsible for K^+^-coupled Cl^−^ outward transport in central neurons. In contrast, the Na^+^–K^+^–2Cl^−^ cotransporter (NKCC) family is responsible for transporting Cl^−^ into cells through a Na^+^–K^+^ -coupled Cl^−^ inward transport. The high intracellular [Cl^−^] that makes GABA_A_ receptors excitatory in developing fetal brains has been linked to an upregulation of *NKCC1* mRNA expression, whereas the low intracellular [Cl^−^] in mature neurons is attributable to an upregulation of *KCC2* mRNA expression (Delpire, 2000).

Additional mechanisms may influence anion gradients across neural membranes. One is the voltage-gated Cl^−^ channel 2 (ClC2), which has an important role in determining the intracellular [Cl^−^] through chloride extrusion in neurons expressing inhibitory GABA_A_ receptors and directly reducing excitability (Ratté and Prescott, 2011). The anion exchanger 3 (AE3) affects both [Cl^−^] and [HCO_3_^−^] by exchanging Cl^−^ and HCO_3_^−^ over cell membranes, while the Na^+^-driven anion exchanger (NDAE) can influence intracellular [Cl^−^] through a Na^+^-coupled HCO_3_^−^ outward transport (Romero *et al*., 2000; Casey *et al*., 2009). Finally, intracellular [HCO_3_^−^] is influenced by the rate of hydration of intracellular CO_2_ through the action of carbonic anhydrases (CAs; Lindskog, 1997).

The function of the GABAergic transmission is also affected by the timing of GABA release and clearance in the extracellular space. Extracellular GABA in not subject to enzymatic degradation, but its turnover relies on diffusion and uptake by specific GABA transporters, GAT1–3 (reviewed by Scimemi, 2014). GABA is susequently processed in the neurons by GABA aminotransferase (GABAT) and glutamate decarboxylases (GAD1–2, also known as GAD67 and GAD65; Delpire, 2000). The clustering, targeting and degradation of the GABA_A_ receptor in the post-synaptic area is regulated by GABA_A_ receptor-associated proteins (GABARAP and GABARAPL; Nemos *et al*., 2003). Both proteins belong to a microtubule-associated protein family.

Interestingly, in some fish the behavioural dysfunctions observed in hypercapnia set only in after several days of exposure to high CO_2_ and then persist for several days after normal CO_2_ levels have been restored (Munday *et al*., 2010). This led Lai *et al*. (2015) to propose that gene transcription may be involved. This could include the expression of GABA_A_ receptor genes and the genes encoding for proteins responsible for establishing Cl^−^ and HCO_3_^−^ ion gradients over neuronal membranes.

The three-spined stickleback (*Gasterosteus aculeatus*) should provide a good model for investigating the effects of CO_2_ on gene expression because its genome has been sequenced and annotated (Kingsley, 2003). Importantly, sustained high-CO_2_ exposure has been shown to alter three-spined stickleback behaviour (Jutfelt *et al*., 2013; Näslund *et al*., 2015), and this impairment can be reversed by treatment with the GABA_A_ antagonist gabazine (Lai *et al*., 2015). Thus, as in other fishes, the neural effects of high-CO_2_ exposure on three-spined stickleback appear to depend on altered GABA_A_ receptor function.

We hypothesized that the proposed ion disturbances leading to altered GABA_A_ receptor function in brains of hypercapnic fish lead to alterations in the expression of genes related to the function of these systems. Consequently, we have quantified the mRNA transcription levels of 56 genes involved in GABAergic transmission and anion regulation in brains of three-spined stickleback exposed to present and predicted future CO_2_ levels. Our analysis included the expression of 28 genes encoding for the GABA_A_ receptor subunits in three-spined stickleback, six for GABA transporters (GAT1–3), GABA aminotransferase (GABAT), three for glutamate decarboxylases (GAD1–2), three for GABA_A_ receptor-associated protein and protein-like (GABARAP and GABARAPL), 14 for ion cotransporters (KCCs, NKCCs, ClC2s, AE3 and NDAE) and two for carbonic anhydrases (CAII and CAVII).

## Materials and methods

### Experimental animals

One hundred marine female three-spine sticklebacks weighing 1.24 ± 0.07 g were caught in Fiskebäckskil, Sweden, during July–August 2012 and were randomly distributed into ten 25 litre glass aquaria of 10 individuals each in Sven Lovén Centre for Marine Sciences, Kristineberg, Sweden. The aquaria were constantly supplied with water at 17.6 ± 1.2°C (SD) and salinity 24.2 ± 3.4 PSU (SD). Chemical parameters such as salinity, oxygen saturation, temperature and pCO_2_ were measured daily, and alkalinity was measured weekly. Further details are given by Jutfelt *et al*. (2013), who published behavioural data from the same groups of fish.

The fish were divided into two experimental groups (distributed in duplicate aquaria for each group), where one group was exposed to increased pCO_2_ (991.3 ± 56.6 μatm), while the other served as a control and was exposed to present-day CO_2_ levels (333.0 ± 30.0 μatm pCO_2_; Jutfelt *et al*., 2013). Fish were kept in a 14 h–10 h light–dark cycle and fed *ad libitum* twice daily with frozen *Artemia* nauplii. The exposures lasted for 43 days. Upon termination of exposure and behavioural studies, 12 individuals weighing 2.06 ± 0.14 g from the control group and 12 individuals weighing 1.58 ± 0.18 g from the CO_2_ group were killed using an overdose of 2-phenoxyethanol in seawater. For the gene expression analysis, the whole brains were rapidly dissected, snap-frozen in liquid nitrogen and stored at −80°C until further use. Prior to downstream experiments, samples were transferred on dry ice to the Department of Biosciences, University of Oslo, Norway.

Animal experiments were carried out in accordance with national regulations and were approved by the ethical committee on animal experiments of Gothenburg, Sweden (ethical permit: Fredrik Jutfelt 100-2010 and 151-2011).

### Quantification of mRNA expression using qPCR

#### RNA extraction and cDNA synthesis

Total RNA was extracted from brains using 15 µl/mg TRIzol^®^ reagent (Invitrogen, Carlsbad, CA, USA). A NanoDrop 2000 UV-Vis Spectrophotometer (Thermo Fisher Scientific, Rockland, DE, USA) and a 2100 BioAnalyzer with RNA 6000 Nano Lab Chip Kit (Agilent Technologies, Palo, Alto, CA, USA) were used to assess the quantity and quality of the extracted total RNA. Prior to cDNA synthesis, 1 µg of total RNA was treated with TURBO DNase using TURBO DNAse-free kit (Ambion Applied Biosystems, Foster City, CA, USA) to avoid any remnants of genomic DNA. Subsequently, cDNA was synthesized in duplicate from each total RNA sample using SuperScript III reverse transcriptase (Invitrogen) and oligo(dT)_18_ in a total reaction volume of 20 µl. All procedures were carried out in accordance with the manufacturer's protocols.

#### Real-time RT-PCR primer design

To our knowledge, expression analyses of the GABA_A_ subunits or genes linked with the GABA_A_ activity studied here have previously not been described in stickleback. Therefore, a total of 56 gene-specific real-time rt-PCR (qPCR) primer pairs were designed from stickleback gene sequences retrieved from the Ensembl database (http://www.ensembl.org/index.html; see Table 1 for accession numbers). For each transcript, a minimum of three primer pairs were initially designed for each nucleotide sequence using Primer3 (http://primer3.ut.ee) and synthesized by ThermoScientific (Ulm, Germany). Emphasis was put on designing primers spanning exon–exon junctions to avoid amplification of any remnant genomic DNA. All primers were analysed for crossing point (Cp) values, primer efficiencies (E) and melting peaks, and their products were sequenced by GATC (Cologne, Germany), ensuring amplification of a single amplicon. The primer pairs showing the highest efficiency, lowest crossing point value and a single melting peak curve were selected for qPCR and are listed in Table 1.

**Table 1:** Primer sequences for qPCR in three-spined stickleback

Gene	GenBank ID		Primers for real-time PCR			
(**A**)						
					E	Cp
Ubiquitin	ENSGACG00000008021	ubc	F	AGACGGGCATAGCACTTGC	1.894 ± 0.001	22.17 ± 0.06
			R	CAGGACAAGGAAGGCATCC		
Ribosomal protein L13A	ENSGACT00000012382	rpl13A	F	CACCTTGGTCAACTTGAACAGTG	1.897 ± 0.006	21.86 ± 0.16
			R	TCCCTCCGCCCTACGAC		
GABA_A_α1 (1of2)	ENSGACT00000027474	α1_1_	F	GGCAGAGTGTGGATTCTGGT	1.896 ± 0.003	25.62 ± 0.11
			R	GGACGGACTCTCTGTTGAGC		
GABA_A_α1 (2of2)	ENSGACT00000027475	α1_2_	F	GCTATGACAATCGCCTCAGG	1.878 ± 0.000	26.75 ± 0.17
			R	TTGTGGAAGAAGGTGTCGGG		
GABA_A_α2	ENSGACT00000024778	α2	F	GAGGATTTCCCCATGGACTT	1.910 ± 0.002	28.14 ± 0.21
			R	CTCCTTCCACCTCCACAGAG		
GABA_A_α3	ENSGACT00000026865	α3	F	CACCCTGAGCATCAGTGCTA	1.846 ± 0.002	32.11 ± 0.27
			R	CGTCGACGATTCTCTTCTCC		
GABA_A_α4	ENSGACT00000024781	α4	F	TTTTGGACCGACTTCTGGAC	1.882 ± 0.001	31.19 ± 0.19
			R	ATTTCCACATCCGAGACAGG		
GABA_A_α5 (1of2)	ENSGACT00000018222	α5_1_	F	TCCCGCCTCAATCAATACCA	1.865 ± 0.003	31.73 ± 0.22
			R	CGGCATGTAGGTCTGGATGA		
GABA_A_α5 (2of2)	ENSGACT00000019800	α5_2_	F	ATGCCTATCCGGTGTCAGAG	1.877 ± 0.001	28.92 ± 0.13
			R	TCAGGTAGAAGTGGGCCATC		
GABA_A_α6a	ENSGACT00000024057	α6a	F	GGTCCATTTCCACCTGCAGA	1.838 ± 0.006	33.83 ± 0.03
			R	GCTCAAGGTGGTCATGGTCA		
GABA_A_α6b	ENSGACT00000027476	α6b	F	CGCCTGATGAACTTCCCCAT	1.898 ± 0.001	26.48 ± 0.22
			R	GACACCGTCTGACCGATGAG		
GABA_A_β1	ENSGACT00000017426	β1	F	GGCGTGGAAAACATTGAACT	1.885 ± 0.005	27.08 ± 0.17
			R	AGACCCAGGACAAGATGGTG		
GABA_A_β2 (1of2)	(i) ENSGACT00000024053	β2_1i_	F	AAGATGAGACCCGACCCCAA	1.906 ± 0.000	27.42 ± 0.14
			R	TGCTCGCCTAGTCCTAATGC		
	(ii) ENSGACT00000024054	β2_1ii_	F	ATCCCGAAACCGCCTCAAAA	1.894 ± 0.003	27.68 ± 0.14
			R	CCTGTCCACGGTTTCCTTCA		
GABA_A_β2 (2of2)	ENSGACT00000027477	β2_2_	F	CGCTGCTTGTATGATGGACC	1.907 ± 0.009	29.63 ± 0.52
			R	AGGCAGTTCGATCTTGTCCA		
GABA_A_β3 (1of2)	(i) ENSGACT00000018209	β3_1at_	F	AGGGATACGACATCCGTCTG	1.811 ± 0.002	34.34 ± 0.20
	(ii) ENSGACT00000018213		R	CGTAGGCCAGTCTCTTGTCC		
GABA_A_β3 (2of2)	(i) ENSGACT00000019821	β3_2at_	F	ACGTACATGCCATCGATCCT	1.853 ± 0.000	28.37 ± 0.04
	(ii) ENSGACT00000019826		R	GGTGTTGATCGTGGTCATCG		
	(iii) ENSGACT00000019833					
GABA_A_γ1	ENSGACT00000026662	γ1	F	ATCAATTACCGGTGGCAGAG	1.820 ± 0.000	29.71 ± 0.09
			R	GGAGACCCAAGACAAGACCA		
GABA_A_γ2	(i) ENSGACT00000027471	γ2at	F	GACAAACCAAGAAGGGCAAA	1.874 ± 0.001	29.59 ± 0.10
	(ii) ENSGACT00000027472		R	GGCACAATGTTGGTCATCTG		
	(iii) ENSGACT00000027473					
GABA_A_γ3 (1of2)	ENSGACT00000018227	γ3_1_	F	GCTGTCTGTCCTTCTCACCT	1.820 ± 0.003	33.08 ± 0.11
			R	CGCAGCTTCTTGTCGTACTC		
GABA_A_γ3 (2of2)	ENSGACT00000019780	γ3_2_	F	GGCTCCGAAACACAACAGAT	1.858 ± 0.014	31.51 ± 0.28
			R	ATGGTGAAGTAGCCCATTCG		
GABA_A_δ	ENSGACT00000007260	δ	F	CTGGAGCTCTCCCAGTTCAC	1.917 ± 0.003	25.02 ± 0.16
			R	GCAGGATGGAAGGCATGTAT		
GABA_A_π (1of2)	ENSGACT00000003740	π_1_	F	TTCTGCCTCCCACCATTCAT	1.835 ± 0.002	32.40 ± 0.31
			R	TTGTTGCCCTCGAAACCAAG		
GABA_A_π (2of2)	ENSGACT00000024472	π_2_	F	AGGCCATCGATGTTTACCTG	1.883 ± 0.003	32.33 ± 0.13
			R	CGAAGCTCCCTGTGTAGGTC		
GABA_A_ρ1 (1of2)	ENSGACT00000012171	ρ1_1_	F	GTCACTGTTACCGCCATGTG	1.830 ± 0.002	29.3 ± 0.15
			R	TGGTGGTGTGGAATTTCTGA		
GABAAρ1 (2of2)	(i) ENSGACT00000016158	ρ1_2at_	F	CACTAAAGTCTGGGGTCCGA	1.896 ± 0.008	33.61 ± 0.03
	(ii) ENSGACT00000016168		R	TTGGTGTTGCTCTTGAAGGC		
GABA_A_ρ2a (1of2)	ENSGACT00000016151	ρ2a_1_	F	GGCAGCCTGTAACATGGACT	1.877 ± 0.001	30.68 ± 0.08
			R	CGTGGTGTGGAACTTCTGGA		
GABA_A_ρ2a (2of2)	ENSGACT00000017273	ρ2a_2_	F	GCATGCAACATGGATTTCAG	1.888 ± 0.001	32.5 ± 0.09
			R	GGATGAGGAACTGGGACAGA		
GABA_A_ρ3a	(i) ENSGACT00000027385	ρ3a_at_	F	AGCAGTACGGAGAGAACACC	1.894 ± 0.001	32.9 ± 0.28
	(ii) ENSGACT00000027384		R	GCATTGCAAAGTCGTGGTCT		
GABA_A_ρ3b	ENSGACT00000002161	ρ3b	F	CTTCATCCACGACACCACCA	1.885 ± 0.001	30.46 ± 0.07
			R	GGGAAGCTGCTGAAGTCCAT		
(**B**)						
Ubiquitin	ENSGACG00000008021	ubc	F	AGACGGGCATAGCACTTGC	1.904 ± 0.003	22.08 ± 0.01
			R	CAGGACAAGGAAGGCATCC		
Ribosomal protein L13A	ENSGACT00000012382	rpl13A	F	CACCTTGGTCAACTTGAACAGTG	1.902 ± 0.010	22.07 ± 0.26
			R	TCCCTCCGCCCTACGAC		
GAT1_1_	ENSGACG00000009684	GAT1_1_	F	CAGTGCAGATGGTTCCCCTC	1.8794 ± 0.000	25.19 ± 0.04
			R	GCGGGGTTCTGATTCTGGTT		
GAT1_2_	(i) ENSGACT00000020044	GAT1_2at_	F	AGAGTACGTGTTCCCAGCATG	1.879 ± 0.003	26.21 ± 0.00
	(ii) ENSGACT00000020046		R	ATAGGTTCGCTGCGTTGGTC		
GAT2_1_	(i) ENSGACT00000004780	GAT2_1at_	F	TGCGTTGATCAAGTACTCTCCT	1.894 ± 0.000	26.97 ± 0.14
	(ii) ENSGACT00000004778		R	CTTGGTTTTCGGCAAGTCGG		
GAT2_2_	ENSGACT00000025159	GAT2_2_	F	TGTCTGCATTGCTTGGGTCT	1.891 ± 0.000	29.62 ± 0.09
			R	AATCGCATAACCCCACCAGG		
GAT2_3_	(i) ENSGACT00000001890	GAT2_3at_	F	GGTCTGGAAGCCCTCGTAAC	1.920 ± 0.002	28.40 ± 0.08
	(ii) ENSGACT00000001897					
	(iii) ENSGACT00000001899		R	GAGAGTCATCCCACTGCAGG		
GAT3	(i) ENSGACT00000009625	GAT3_at_	F	GCGGGATGTGTTTGCTGTTT	1.901 ± 0.003	26.51 ± 0.03
	(ii) ENSGACT00000009632		R	CCAGTCAGGGTAGGTGTACA		
GABAT	(i) ENSGACT00000006245	GABAT_at_	F	TGTCCGATCCAAGCAGTCTG	1.855 ± 0.005	27.73 ± 0.04
	(ii) ENSGACT00000006238		R	CATTGTCTGAACCCGGGACA		
GAD1a	ENSGACT00000006685	GAD1a	F	GGGACACCTTGAAGTACGGA	1.858 ± 0.001	30.74 ± 0.02
			R	CATGAGCACAAAGACAGGGG		
GAD1b	ENSGACT00000017175	GAD1b	F	CCATTGGGTTTGAGCAGCAC	1.891 ± 0.002	25.93 ± 0.09
			R	CATGTCTCTCAGGCTGGGTG		
GAD2	ENSGACT00000006820	GAD2	F	ACCTCTCTTCGCCATAACCG	1.876 ± 0.005	30.15 ± 0.15
			R	ATCATCTTGTGCGGGTTCCA		
GABARAP	(i) ENSGACG00000025686	GABARAP_at_	F	ATATCTCGTCCCCTCCGACC	1.913 ± 0.003	23.05 ± 0.09
	(ii) ENSGACG00000025685		R	CGCTCTCATCACTGTAGGCA		
GABARAPL1	ENSGACG00000013851	GABARAPL1	F	AGGTGAGGAGAGCAGAAGGA	1.933 ± 0.000	24.74 ± 0.12
			R	GGGGAAGGGAGTTGTTGACA		
GABARAPL2	(i) ENSGACG00000002829	GABARAPL2_at_	F	AAGTACCTGGTGCCCTCTGA	1.913 ± 0.007	26.72 ± 0.05
	(ii) ENSGACG00000002816					
	(iii) ENSGACG00000002836		R	TTTTCGTACAGCTGCCCCAT		
(**C**)						
Ubiquitin	ENSGACG00000008021	ubc	F	AGACGGGCATAGCACTTGC	1.894 ± 0.001	22.17 ± 0.06
			R	CAGGACAAGGAAGGCATCC		
Ribosomal protein L13A	ENSGACT00000012382	rpl13A	F	CACCTTGGTCAACTTGAACAGTG	1.897 ± 0.006	21.86 ± 0.16
			R	TCCCTCCGCCCTACGAC		
KCC1	ENSGACT00000022002	KCC1	F	ACAACGGAGAGCCTACATGG	1.844 ± 0.001	30.21 ± 0.24
			R	TCATGCCTAGGAAGGACAGC		
KCC2a	ENSGACT00000007272	KCC2a	F	AGAGCAGAACGTGGAACAGC	1.803 ± 0.001	29.42 ± 0.44
			R	GCACGCTGAGACTGTTCGTA		
KCC2b	ENSGACT00000003694	KCC2b	F	AGAACATCTCCAGCTACCCG	1.909 ± 0.030	32.59 ± 0.07
			R	CGCAGGTGATAGAGGAAGGT		
KCC3	ENSGACT00000025029	KCC3	F	GGCGCTCATGTTCATATCCT	1.901 ± 0.001	30.93 ± 0.17
			R	GCGTCCTCGTCCAGTTTTAG		
KCC4a	ENSGACT00000019164	KCC4a	F	GCCAAGAACATCGACCATTT	1.903 ± 0.003	32.52 ± 0.16
			R	CACCACAGCATCCAGACGTA		
KCC4b	(i) ENSGACT00000001353	KCC4b	F	AAAGACACAGAGGCCAGGAA	1.864 ± 0.003	31.23 ± 0.20
	(ii) ENSGACT00000001355		R	CCATGAGGATTGTGTTGTGC		
NKCC1 (1of2)	ENSGACT00000019494	NKCC_1i_	F	TCCGAATCCTGTCCCTCCAA	1.831 ± 0.000	27.86 ± 0.00
			R	ATGGTTCCTTTGCCCTGCTT		
	ENSGACT00000019488	NKCC_1ii_	F	TTAAACTCCCCGCGATGCTT	1.858 ± 0.000	32.56 ± 0.34
			R	GTTGTCGGTGATCCTCCAGG		
NKCC1 (2of2)	(i) ENSGACT00000024304	NKCC_2at_	F	GAGTCTTGGCCCAGAGTTTG	1.915 ± 0.004	29.28 ± 0.36
	(ii) ENSGACT00000024305		R	GCGGATATCGTTGAGTTCGT		
ClC2 (1of3)	(i) ENSGACT00000000266	ClC21_at_	F	CCAGAGAAAGAAGGCCTGGA	1.919 ± 0.004	26.77 ± 0.01
	(ii) ENSGACT00000000267		R	CATCCTCCACATCTGCGTCG		
ClC2 (2of3)	ENSGACT00000013852	ClC2_2_	F	TTAAAACACGGTTCCGGCTC	1.886 ± 0.001	29.34 ± 0.16
			R	ATCAGCCGGTTCAGGTAGAC		
ClC2 (3of3)	ENSGACT00000017906	ClC2_3_	F	GGCCAAAGTCATCGGTCTGA	1.884 ± 0.002	30.28 ± 0.19
			R	CCACCGAAAAGAGGAGCCAT		
NDAE	ENSGACT00000000854	NDAE	F	TCCTCATGTGTGCGTTCCTC	1.867 ± 0.001	30.47 ± 0.28
			R	AACCTCGGTCGTCTCTGGTA		
AE3	ENSGACT00000003278	AE3	F	GGAGCAATTATGACCTGCGG	1.865 ± 0.001	30.65 ± 0.12
			R	GACACCGCGATGACTTCTTC		
CAII	ENSGACT00000006681	CAII	F	CTGACTTCGACCCTTCCACC	1.910 ± 0.000	24.94 ± 0.14
			R	GCAGCTCGCGGAATTTCTTC		

Primer sequences used for qPCR: (A) GABA_A_ receptor subunits qPCR primers; (B) GAT, GABAT, GAD, GABARAP and GABARAPL qPCR primers; and (C) Ion cotransporters qPCR primers. Abbreviations: F, forward primer; and R, reverse primer. The lower case number and/or letter represents a paralogue sequence and/or splice variance, respectively: _1_, paralogue 1; _2_, paralogue 2; _i_, splice variant 1; _ii_, splice variant 2; and _at_, primer pair that does not discriminate between splice variants. Priming efficiencies (E) and crossing point (Cp) values are given in the two rightmost columns. Values are means ± SEM.

For genes with known paralogues or splice variants, efforts were made to design transcript-specific primers when possible, in order to discriminate between closely related transcripts. A comparison aiming at determining identities between genes was carried out using a global alignment (NCBI-Needleman-Wunsch Global Align Nucleotide Sequences; blast.ncbi.nlm.nih.gov), which is a sequence alignment method based on the Needleman–Wunsch algorithm (Needleman and Wunsch, 1969) used to find the best optimal alignment along two sequences (Table S1).

Thirty-five gene sequences for GABA_A_ receptor subunits were retrieved from the Ensemble stickleback database. Diverse paralogue sequences exist in the GABA_A_ subunit families, except for the δ subunit, which has only one known gene variant (see Table 1A). The majority of these sequences showed a distant relationship (identitites ranging from 37 to 78%; Table S1A–E), and qPCR primers were directed at conserved regions. In contrast, paralogues belonging to the subunits β3, γ2 and ρ1 showed a close identity (51–100%), and qPCR primers were directed at poorly conserved regions and analysed as single transcripts (Table S1B, C and E). Among all paralogues, Ensembl presents alternative splice variants for β2_1_, β3_1_ and β3_2_ (Table 1A). In the γ family, γ2 is the only subunit that splices for three alternative transcripts (Table 1A), and for the ρ subunits, two alternative variants are known to be present for ρ1_2_ and ρ3a: ρ1_2i_, ρ1_2ii_ and ρ3a_i_, ρ3a_ii_ (Table 1A). Altogether, a total of 28 qPCR primers were designed for the gene expression analysis of subunits (some sequences showed too much similarity to allow for the design of specific primers).

For the genes involved in GABA turnover, 11 different gene paralogues were found to encode for GAT, three for GAD, one for GABAT, two for GABARAP and four for GABARAPL. We designed a total of 13 qPCR primers, of which some will work for more than one transcript. Moreover, four different genes encoding for ClC2, seven for KCCs and four for NKCC1 are found in the stickleback genome, whereas AE3, NDAE and CAII have only one variant (Table 1). A total of 15 qPCR primers were designed for the ion cotransporter analysis. Effort was made to design primers able to detect CAVII, but we were unsuccessful in detecting this transcript.

As for the GABA_A_ subunits, splice variants are present for some of the members of GAT and GAD families (Table 1B). The gene GAT1 splices for two splice variants (Table 1B). The other transporters, GAT2_1_, GAT2_3_ and GAT3, splice into two (GAT2_1i_ and GAT2_1ii_), three (GAT2_3i_, GAT2_3ii_ and GAT2_3iii_) and two variants (GAT3_i_ and GAT3_ii_), respectively (Table 1B). The GABARAPL2 gene encodes for three splice variants, GABARAPL2i and GABARAPL2ii (Table 1B). In the KCC family, two KCC4b splice variants are known, KCC4b_i_ and KCC4b_ii_ (Table 1C). Likewise for ClC2_1_, there are two alternative splice variants (ClC2_1i_ and ClC2_1ii_; Table 1C). Two NKCC1 paralogues exist (NKCC1_1_ and NKCC1_2_), both having alternative splice variants (Table 1C).

#### Quantitative PCR

Quantitative PCR was carried out in duplicates using 1:30 diluted cDNA (3 μl), LightCycler 480 SYBR Green I Master Mix (5 μl; Roche Diagnostics, Basel, Switzerland), primers (1 μl; 5 μM) and nuclease-free water (1 μl; Ambion Applied Biosystems). The reaction mix and samples were loaded onto 384 multiwell plates (Roche Diagnostics) using an Agilent Bravo robot (Agilent Technologies, USA) The following qPCR program was used: (i) 95°C for 10 min; (ii) 95°C for 10 s; (iii) 60°C for 10 s; (iv) 72°C for 13 s; and (v) repeat steps (ii) to (iv) 42 times. A melting curve analysis was performed for each amplicon after the qPCR program. Ubiquitin (ubc) and ribosomal protein L13A (rpl13A) were used as reference genes for normalization, as they have previously been demonstrated to be the most stably expressed genes in the three-spined stickleback (Hibbeler *et al*., 2008) (Table 1). The geometric average of their expression was used to normalize the data sets, because this method has been shown to be a prerequisite for an accurate qPCR expression analysis leading to the possibility of studying small expression differences (Vandesompele *et al*., 2002; Hellemans *et al*., 2007).

The Cp values and priming efficiencies for each reaction were calculated using the second derivative maximum method (Roche Lightcycler 480; Rasmussen, 2001) and the LinRegPCR software (Ruijter *et al*., 2009), respectively. Subsequently, relative mRNA expression levels were calculated using the following formula:

$$\mathrm{Expression}\;\mathrm{of}\;\mathrm{target}\;\mathrm{gene}=\left({E_{ga}}^{\wedge }C_{pga}/{E_{tar}}^{\wedge }C_{ptar}\right)$$

Where *ga* is the geometric average of the two reference genes; *tar* is the gene of interest, *E* is priming efficiency and *Cp* is the crossing point.

Given that duplicate cDNA syntheses were performed, and each of these were analysed in duplicates in the qPCR analyses, four data points were present for each original sample for each primer pair used, and their means were used in the mRNA expression calculations.

### Statistical analysis

All statistical analyses were performed using GraphPad Prism (GraphPad Software; version 6.0d; Mac OS X). Normality and homogeneity of variance were assessed using the D'Agostino & Pearson omnibus normality test and *F*-test. According to function, data were grouped into seven families as follows: (i) GABA_A_ α subunits; (ii) GABA_A_ β subunits; (iii) GABA_A_ γ subunits; (iv) GABA_A_ δ, π and ρ subunits; (v) GAT, GAD and GABAT; (vi) GABARAP and GABARAPL; and (vii) KCC, NKCC, ClC2, AE3, NDAE and CAVII. Two-way analysis of variance (ANOVA) followed by the Sidak *post hoc* test was used to examine differences in expression between the genes within the families and between the two treatment groups. A value of *P* < 0.05 was considered significant. All data are presented as means ± SEM, unless otherwise stated.

## Results

**Figure 1:**
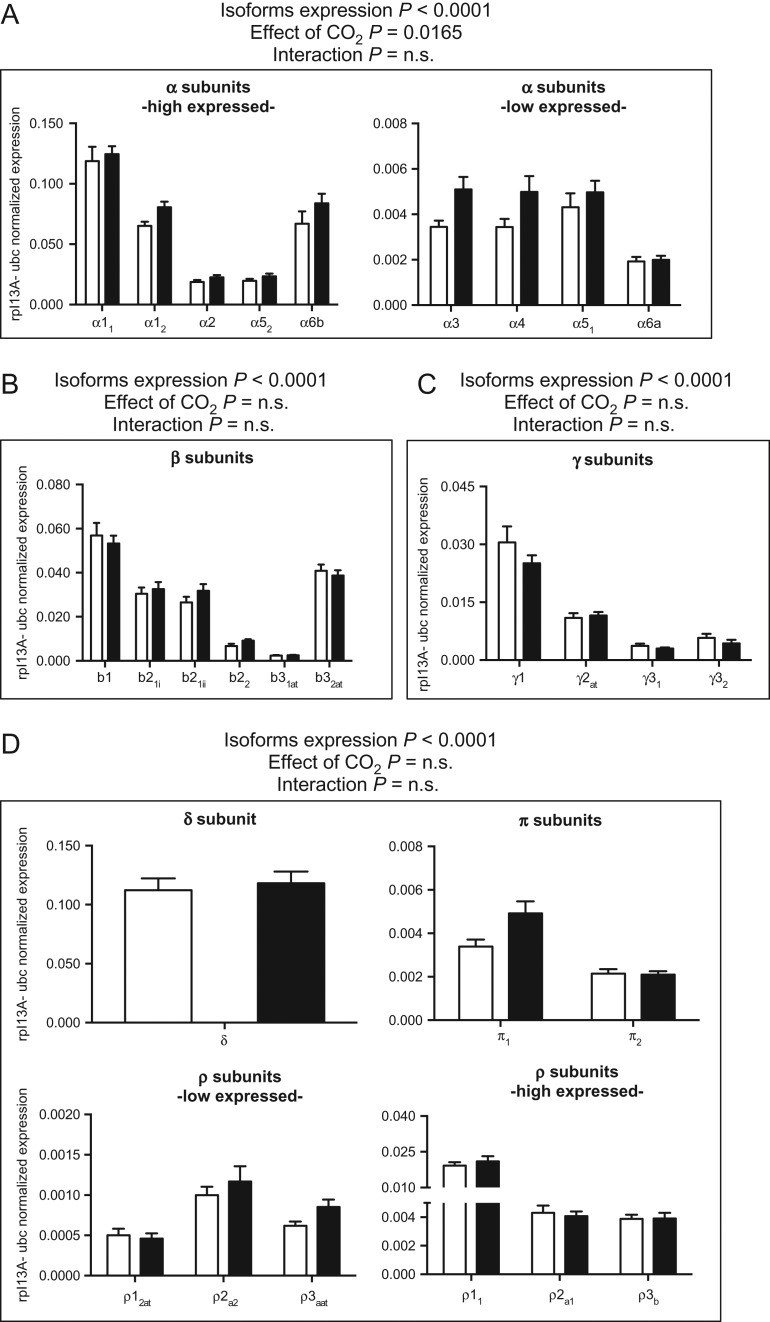
Messenger RNA expression levels of GABA_A_ receptor subunits. Data were normalized to the geometric average of the reference genes ribosomal protein L13A (rpl13A) and ubiquitin (ubc) and grouped into four families as follows: α subunits (**A**); β subunits (**B**); γ subunits (**C**) and δ, π and ρ subunits (**D**). Each family was analysed by two-way ANOVA followed by Sidak *post hoc* test. Open and filled columns represent three-spined sticklebacks exposed to control water (*n* = 12) and high-CO_2_ water (*n* = 12) for 43 days. Values are shown as means + SEM.

As listed in Table 1A, GABA_A_ subunits can be regrouped into six families: α(1–6), β(1–3), γ(1–3), δ, π and ρ(1–3). In contrast to mammals, no genes encoding for ε and θ subunits are present in the stickleback genome. All GABA_A_ paralogues retrived on the Ensembl database were found to be expressed in the three-spined stickleback brain (Fig. 1A–D). Expression within each gene family was analysed using two-way ANOVA, with subunit and CO_2_ treatment as the two variables. Not surprisingly, the mRNA transcripts levels differed significantly for the different subunits (Fig. 1; two-way ANOVA, *P* < 0.001). The most highly expressed GABA_A_ subunits in the three-spined stickleback belonged to the α, β, γ and δ families (Fig. 1A–D), and within these families the expression was dominated by α1_1_ and α1_2_, α6b, β1, γ1 (there was only a single isoform for the δ subunit). Among the ρ subunits, ρ1_1_ was the most abundant (Fig. 1D). The π subunits showed the lowest expression levels (Fig. 1D). Exposure of three-spined stickleback to elevated pCO_2_ (~990 μatm) resulted in significantly altered expression levels for relatively few of the GABA_A_ subunits investigated. The α family subunits, which are composed of nine different isoforms, showed a significantly higher expression in high-CO_2_ fish compared with control fish (Fig. 1A; two-way ANOVA, *P* = 0.0165). All α subunits showed a numerically higher mean expression in the high-CO_2_ group, with increased mRNA transcription levels of 24, 48, 50 and 25% for the α1_2_ α3, α4 and α6b, respectively, although the *post hoc* test failed to identify significant treatment effects for any individual isoform (Fig. 1A; Sidak *post hoc* test, *P* > 0.05). The high-CO_2_ exposure did not significantly affect the expression of the β (two-way ANOVA, *P* = 0.6639), γ (two-way ANOVA, *P* = 0.1861), δ, π or ρ subunits (two-way ANOVA, *P* = 0.7189; Fig. 1B–D).

**Figure 2:**
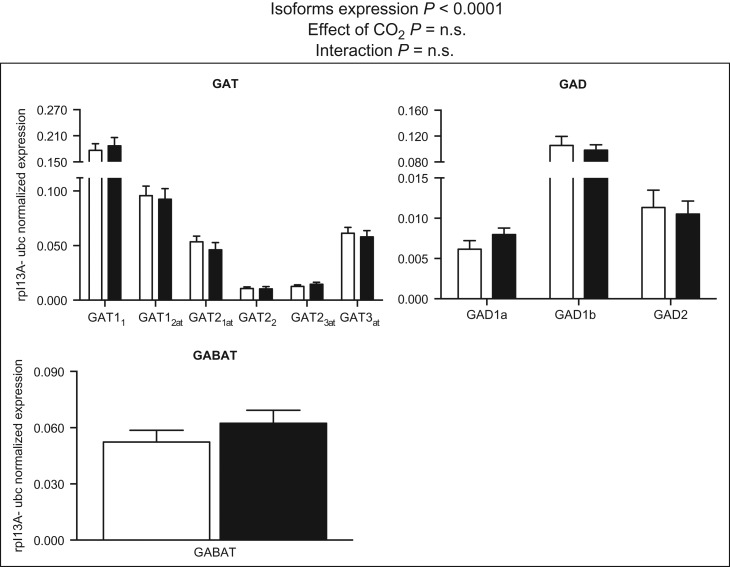
Messenger RNA expression levels of GAT, GABAT and GAD genes. Data were normalized to the geometric average of the reference genes ribosomal protein L13A (rpl13A) and ubiquitin (ubc). Data were analysed by two-way ANOVA followed by Sidak *post hoc* test. Open and filled columns represent three-spined sticklebacks exposed to control water (*n* = 12) and high-CO_2_ water (*n* = 12) for 43 days. Values are shown as means + SEM.

**Figure 3:**
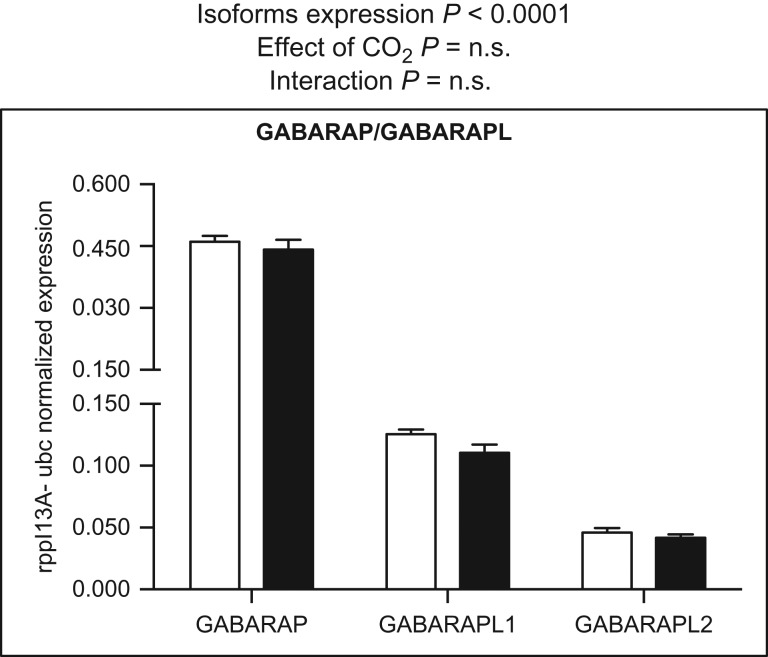
Messenger RNA expression levels of GABARAP and GABARAPL genes. Data were normalized to the geometric average of the reference genes ribosomal protein L13A (rpl13A) and ubiquitin (ubc). Data were analysed by two-way ANOVA followed by Sidak *post hoc* test. Open and filled columns represent three-spined sticklebacks exposed to control water (*n* = 12) and high-CO_2_ water (*n* = 12) for 43 days. Values are shown as means + SEM.

Of the genes involved in GABA turnover included in Ensembl Genome Browser (GAT, GAD, GABAT, GABARAP and GABARAPL), all were found to be expressed in three-spined stickleback brain (Figs 2 and 3). Within families, there were significant differences in the mRNA abundance of the paralogue members (two-way ANOVA, *P* < 0.001). In the control group, the GAT1 paralogues (GAT1_1_ and GAT1_2at_) were more abundant than GAT2_1–3_ (Fig. 2). In the GAD family, GAD1b displayed higher mRNA expression levels than the GAD1a and GAD2 transcripts (Fig. 2). GABARAP was almost four times more highly expressed than GABARAPL1 and eight times more highly expressed than GABARAPL2 (Fig. 3). In our experiment, none of the transporters or enzymes involved in GABA metabolism displayed significant alterations in expression in response to high-CO_2_ treatment (Figs 2 and 3; two-way ANOVA, *P* > 0.05).

**Figure 4:**
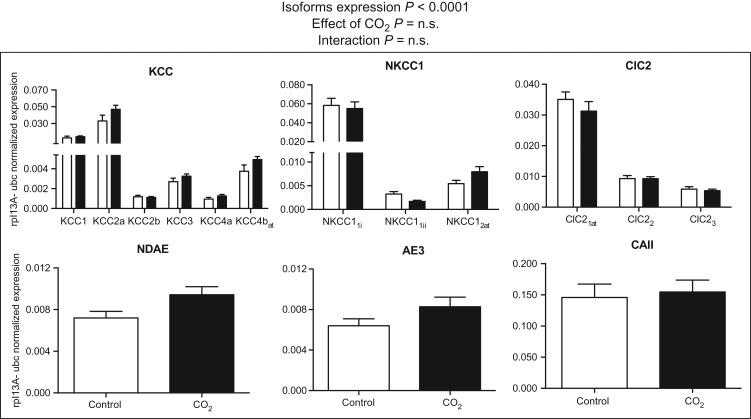
Messenger RNA expression levels of KCCs, NKCC1, ClC2, NDAE, AE3 ion cotransporters and CAII enzyme. Data were normalized to the geometric average of the reference genes ribosomal protein L13A (rpl13A) and ubiquitin (ubc). Data were analysed by two-way ANOVA followed by Sidak *post hoc* test. Open and filled columns represent three-spined sticklebacks exposed to control water (*n* = 12) and high-CO_2_ water (*n* = 12) for 43 days. Values are shown as means + SEM.

Of the genes involved in ion transport, the expression level of the transcripts differed between the members of the gene families (two-way ANOVA, *P* < 0.0001; Fig. 4). KCC2a, ClC2_1at_ and NKCC1_1i_ were the most highly expressed transcripts among the ion transporters (Fig. 4). Exposure to high CO_2_ did not cause significant changes in mRNA expression levels for the ion transporter transcripts in high-CO_2_-treated fish compared with control fish (Fig. 4; two-way ANOVA, *P* > 0.05).

## Discussion

Changes in the function of GABA_A_ receptors caused by altered ion gradients have been suggested as a general mechanism behind the behavioural disturbances seen in CO_2_-exposed fish (Nilsson *et al*., 2012; Chivers *et al*., 2014; Chung *et al*., 2014; Hamilton *et al*., 2014; Lai *et al*., 2015). Exposure to high CO_2_ levels triggers acid–base adjustments in fish involving altered levels of Cl^−^ and HCO_3_^−^ (Brauner and Baker, 2009), which have been suggested to change neuronal membrane gradients of these ions, switching some GABA_A_ receptors from being inhibitory to excitatory (Nilsson *et al*., 2012). Based on the scarce data available, calculations of GABA_A_ equilibrium potentials of neurons in fish exposed to near-future pCO_2_ are consistent in showing a possibility for a shift in the GABA_A_ receptor equilibrium potential from causing hyperpolarization to depolarization (Heuer and Grosell, 2014; Nilsson and Lefevre, 2016; Heuer *et al*., 2016; Regan *et al*., 2016). Here, we hypothesized that an increase in CO_2_ levels in the marine environment, triggering acid–base regulatory mechanisms in fish, could lead to changes in the expression of genes involved in regulating GABA_A_ receptor function and neuronal ion distribution. Such molecular changes could be adaptive. However, the persistence of the behavioural disturbances reported in some experiments, and a lack of transgenerational acclimation (Welch *et al*., 2014), suggest that possible molecular responses are insufficient, or even maladaptive.

The present study is the first comprehensive expression analysis focused on genes involved in GABAergic transmission in fish exposed to elevated CO_2_. The fish in this study were the same individuals as those previously examined behaviourally (Jutfelt *et al*., 2013), where the behavioural alterations, including reduced exploratory behaviour and lateralization, were characterized and found to persist for the whole experimental period. Of the 28 GABA_A_ receptor subunits examined herein, there was a significant effect of the high-CO_2_ treatment on the mRNA expression level for the α family subunits, all showing a tendency to be more highly expressed in the CO_2_ group. This could suggest some subunit rearrangement of GABA_A_ receptors in this group, assuming that gene expression is reflected in protein expression. Changes in the GABA_A_ receptor composition are often suggested to induce a change in the receptor function (reviewed by Farrant and Kaila, 2007). The α subunits play important roles on desensitization and deactivation of the receptor, because the GABA binding is presumed to take place at the α–β interfaces (Böhme *et al*., 2004). In the present experiment, changes at the mRNA expression level of the α subunit family after exposure to high CO_2_ might indicate possible compensatory mechanisms used by three-spined stickleback to restore proper GABA_A_ receptor function, but further investigations are required. However, if adaptive, the changes are apparently not sufficient, because the behavioural alterations detected in the same individuals by Jutfelt *et al*. (2013) remained, and if anything increased, during the 43 day exposure period. Also, we cannot exclude the possibility that the changes detected in this study are maladaptive rather than adaptive and contribute to the behavioural alterations.

As mentioned, the three dominant subunits that make up the mammalian receptor are α, β and γ (reviewed by Farrant and Kaila, 2007), and our data show that these subunit families are also highly expressed in three-spined stickleback, but it is striking that the δ subunit is also expressed at a level similar to the most highly expressed α subunits (Fig. 1). Although the mammalian receptors are dominated by subunits α1 β2 and γ2 (Pirker *et al*., 2000), the most predominantly expressed subunits in three-spined stickleback were found to be α1, α6b, β1, γ1 and δ. Receptors that comprise γ2 in association with α1, α2 or α3 subunits are normally localized to post-synaptic membranes, where they mediate a phasic inhibition involving a rapid and brief inhibitory post-synaptic potential in response to GABA in the synaptic cleft (reviewed by Farrant and Nusser, 2005). In contrast, GABA leaking out of the synapse can activate extrasynaptic GABA_A_ receptors made up of δ, α4 or α6 subunits, and these are mainly responsible for slower, but longer-lasting inhibitory post-synaptic potentials, causing tonic inhibition (reviewed by Farrant and Nusser, 2005). Consequently, the high expression of the extrasynaptic δ and the α6b subunits might indicate a more important role of extrasynaptic GABA_A_ receptors in three-spined stickleback compared with mammals. Interestingly, previous studies on crucian carp (*Carassius carassius*) and zebrafish (*Danio rerio*) brain found a dominating expression of the δ subunits (Ellefsen *et al*., 2008; Cocco *et al*., 2016). In light of this high expression of δ subunits, it is tempting to suggest that extrasynaptic GABA_A_ receptors causing tonic inhibition play more important roles in fish than in mammals. In contrast to both crucian carp and zebrafish, three-spined stickleback express alternative splice variants for β2, β3_1_, β3_2_, ρ1_2_ and ρ3a. The only common subunit that exhibits alternative splicing in all three species is γ2 (Ellefsen *et al*., 2008; Cocco *et al*., 2016). The presence of numerous splice transcripts in three-spined stickleback brain could mean that GABA_A_ receptor isoforms are particularly diverse in this species, and fishes differ in the degree to which alternative splicing is used to modulate GABA_A_ receptor function (see also Cresko *et al*., 2003).

Perhaps surprisingly, exposure to high CO_2_ did not result in significant changes in the mRNA expression levels for ion cotransporters. Heuer *et al*. (2016) showed that the increase in plasma partial pressure of CO_2_ (in millimetres of mercury) in spiny damselfish (*Acanthochormis polyacanthus*) kept in high-CO_2_ conditions was accompanied by increases in intracellular and extracellular HCO_3_^−^ concentrations, with an assumed decrease in intracellular Cl^−^ (Heuer *et al*., 2016). Based on their measurements, they calculated a positive deviation in the *E*_GABAA_ resting potential in fish exposed to 1900 µatm CO_2_, consistent with a shift in the GABA action towards depolarization (excitation) rather than hyperpolarization (inhibition). Such alterations could be compensated for by changes in the expression of the Cl^−^ transporters NKCC1 and KCC2 in fish exposed to high CO_2_. As mentioned, these transporters are known to play important roles in setting the reversal potential for Cl^−^ (*E*_Cl_) in the mammalian central nervous system, leading to a shift of the GABA_A_ receptor function from excitatory in immature neurons to inhibitory in mature neurons (Delpire, 2000). In any case, this does not appear to happen in CO_2_-exposed stickleback in the present conditions, as we found no significant changes in the expression of NKCC1 and KCC2, or in other Cl^−^ and HCO_3_^−^ transporters.

Our gene expression results are in agreement with the recent findings of Schunter *et al*. (2016) on juvenile spiny damselfish (*Acanthochromis polyacanthus*). In a transcriptome and proteome analysis, they investigated the molecular responses of offspring of CO_2_-tolerant and CO_2_-sensitive parents reared in control or high-CO_2_ conditions. The main molecular differences between the two groups were found among genes involved in circadian rhythm control, such as *bmal1*, *clock*, *per1* and *nr1d1* (Schunter *et al*., 2016). In contrast, the GABA_A_ receptor genes were expressed at similar levels across treatments. The only possible change seen in the GABAergic system was at the protein level of an enzyme that may participate in GABA synthesis, aldehyde dehydrogenase 9 member 1 (Al9A1), which were more highly expressed in the offspring of the CO_2_-tolerant parents (Schunter *et al*., 2016).

### Conclusions and perspectives

In general, the present findings show that exposure of three-spined stickleback to elevated CO_2_ resulted in only few and minor changes in the expression of genes involved in GABAergic neurotransmission in the brain. If these few adjustments reflect compensatory mechanisms they are apparently not sufficient, because the behavioural dysfunctions remained during the course of the 43 day high-CO_2_ exposure (Jutfelt *et al*., 2013). Thus, the present results, together with results reporting that aberrant behaviours displayed by fish exposed to elevated pCO_2_ are persistent and not reduced even by transgenerational acclimation (Welch *et al*., 2014), lead to the worrying conclusion that fish might be incapable of adaptive responses to these new conditions. Given that globally sustained pCO_2_ levels >500 µatm have probably not occurred on earth during the last 30 million years (Beerling and Royer, 2011), we may have to face the conclusion that many present-day fishes do not possess the genes and mechanisms necessary to cope with the projected near-future elevation of CO_2_ levels.

## Supplementary Material

Supplementary Data
