# Expression of genes involved in brain GABAergic neurotransmission in three-spined stickleback exposed to near-future CO_2_

**DOI:** 10.1093/conphys/cox004

**Published:** 2017-01-20

**Authors:** Floriana Lai, Cathrine E. Fagernes, Fredrik Jutfelt, Göran E. Nilsson

*Conserv Physiol* (2016) 4(1): cow068; doi:10.1093/conphys/cow068

The article has been updated to correct an error in one of the figures. The publisher wishes to apologize for the mistake.

