# Supplementary material for: Expression of genes involved in brain GABAergic neurotransmission in three-spined stickleback exposed to near-future CO2
Source: Conserv Physiol. 2016 Dec 29;4(1):cow068. doi: 10.1093/conphys/cow068 (PMC5196030; doi:10.1093/conphys/cow068)
Supplement: Supplementary Data [file cow068_zebrafish_manus_v12_suppl.docx]

## Supplementary information

## Elevated pCO_2_ affects behavioral lateralization in zebrafish (*Danio rerio*)

Laura Vossen^1^, Fredrik Jutfelt^2^, Arianna Cocco^1^, Per-Ove Thörnqvist^1^ and Svante Winberg^1^

## Statistical analyses

Statistical analyses were performed using R language and environment for statistical computing and graphics, version 3.2.3

(R Development Core Team 2013). To test for an effect of CO_2_ exposure and gabazine treatment on activity in the open field test, linear mixed effects models using REML were fitted to the activity variables, with CO_2_ exposure level, gabazine treatment and sex as fixed effects and exposure tank and number of days exposed as random effects (random intercept models) using the function ‘lmer’ inside the package ‘lme4’ (Bates et al. 2015). The ‘step’ function from the ‘lmerTest’ package was used to perform automated backward selection first of the random effects (likelihood-ratio tests, α=0.10) followed by backward elimination of the fixed part (Wald F tests based on Satterthwaite’s approximation for the denominator degrees of freedom, α=0.05). Using the same fixed and random effects, a generalized linear mixed effects model (GLMM) was fitted to thigmotaxis (the proportion of time spent at the wall), using Laplace approximation and a binomial error distribution with the function ‘glmer’ inside the ‘lme4’ package (Bates et al. 2015). Backward elimination was performed manually here, using likelihood-ratio tests for selection of the random effects and Wald Chisquare tests to select the fixed effects. An observation-level random effect was added to correct for overdispersion. To test whether CO_2_ exposure, gabazine treatment and sex affected the number of homebases, initially a similar GLMM was fitted, but since the random effects were not significant here, the analysis collapsed into a GLM with a poisson error distribution. To test for effects on lateralization, a GLMM similar to the GLMM for thigmotaxis was fitted to the data, the only difference being that the response variable was the proportion of right turns.

**Table S1: Sample sizes in the open field test (OFT).**

|  |  | *p*CO_2_ | |
| --- | --- | --- | --- |
| Treatment | Sex | 400 **μ**atm | 1600 **μ**atm |
| water | males | 12 | 15 |
|  | females | 19 | 15 |
| gabazine | males | 16 | 11 |
|  | females | 13 | 17 |

**Table S2: Sample sizes in the lateralization test.**

|  |  | *p*CO_2_ | |
| --- | --- | --- | --- |
| Treatment | Sex | 400 μatm | 1600 μatm |
| water | males | 8 | 11 |
|  | females | 10 | 7 |
| gabazine | males | 7 | 8 |
|  | females | 9 | 8 |

**Table S3: Results of the linear mixed-effects model (LMM) of swimming speed (in fish lengths per second) as a function of CO_2_ exposure, gabazine treatment and sex. The minimal adequate model only contained exposure time as a random effect.**

|  |  | **var** | **stdev** | **df** | | **Chisq^1^** | | **P** | |
| --- | --- | --- | --- | --- | --- | --- | --- | --- | --- |
| Random effects^1^ | **Exposure time** | **0.04554** | **0.2134** | **1** | | **3.32** | | **0.0685** | |
|  |  | **estimate** | **SE** | **df** | **F^2^** | | **P** | |  |
| Fixed effects^2^ | CO_2_ exposure |  |  | 1, 103 | 0.1393 | | 0.7098 | |  |
|  | **Gabazine treatment** | **-0.0025** | **0.1654** | **1, 105** | **5.7629** | | **0.0181** | |  |
|  | **Sex** | **0.8192** | **0.1780** | **1, 113** | **17.6864** | | **0.0001** | |  |
|  | CO_2_ exposure * Gabazine treatment |  |  | 1,101 | 0.0506 | | 0.8225 | |  |
|  | **Gabazine treatment * Sex** |  |  | **1, 107** | **5.5610** | | **0.0202** | |  |
|  | CO_2_ exposure * Sex |  |  | 1, 107 | 0.2244 | | 0.6367 | |  |
|  | CO_2_ exposure * Gabazine treatment * Sex |  |  | 1, 110 | 0.4774 | | 0.4911 | |  |

^1^Likelyhood-ratio tests, α=0.10.

^2^Wald F tests with type II errors and Satterthwaite’s approximation of the denominator degrees of freedom.

**Table S4: Results of the generalized linear mixed-effects model (GLMM) of thigmotaxis (the proportion of time spent at the wall of the open field arena) as a function of CO_2_ exposure, gabazine treatment and sex.**

|  |  | **df** | **Chisq^1^** | **P** | |  | |  | |
| --- | --- | --- | --- | --- | --- | --- | --- | --- | --- |
| Random effects^1^ | **Observation-level random effect** | **1** | **31945** | **<0.0001** | |  | |  | |
|  | **Exposure time** | **1** | **3.0592** | **0.0803** | |  | |  | |
|  |  | **estimate** | **SE** | **df** | **Chisq^2^** | | **P** | |  |
| Fixed effects^2^ | CO_2_ exposure |  |  | 1 | 1.4987 | | 0.2209 | |  |
|  | **Gabazine treatment** | **-0.1341** | **0.3730** | **1** | **5.2877** | | **0.0215** | |  |
|  | Sex |  |  | 1 | 0.0010 | | 0.9752 | |  |
|  | CO_2_ exposure * Gabazine treatment |  |  | 1 | 0.2797 | | 0.5969 | |  |
|  | CO_2_ exposure * Sex |  |  | 1 | 0.4085 | | 0.5227 | |  |
|  | Gabazine treatment * Sex |  |  | 1 | 1.6228 | | 0.2027 | |  |
|  | CO_2_ exposure * Gabazine treatment * Sex |  |  | 1 | 1.6524 | | 0.1986 | |  |

^1^Likelyhood-ratio tests, α=0.10.

^2^Wald Chisquare tests with type II errors.

**Table S5: Results of the generalized linear mixed-effects model (GLMM) of lateralization (the proportion of right turns) as a function of CO_2_ exposure, gabazine treatment and sex. The minimal adequate model only contained an observation-level random effect.**

|  |  |  |  | **df** | | **Chisq** | | **P** | |
| --- | --- | --- | --- | --- | --- | --- | --- | --- | --- |
| Random effects^1^ | **Observation-level random effect** |  |  | **1** | | **1.9933** | | **0.158** | |
|  |  | **estimate** | **SE** | **df** | **Chisq^2^** | | **P** | |  |
| Fixed effects^2^ | **CO_2_ exposure** | **0.3688** | **0.1290** | **1** | **9.0402** | | **0.0026** | |  |
|  | Gabazine treatment |  |  | 1 | 0.0052 | | 0.9426 | |  |
|  | Sex |  |  | 1 | 0.5490 | | 0.4587 | |  |
|  | CO_2_ exposure * Gabazine treatment |  |  | 1 | 0.3021 | | 0.5826 | |  |
|  | CO_2_ exposure * Sex |  |  | 1 | 1.4515 | | 0.2283 | |  |
|  | Gabazine treatment * Sex |  |  | 1 | 0.9457 | | 0.3308 | |  |
|  | CO_2_ exposure * Gabazine treatment * Sex |  |  | 1 | 0.0174 | | 0.8952 | |  |

^2^Wald Chisquare tests with type II errors.
